# NAIP/NLRC4 inflammasome participates in macrophage responses to *Trypanosoma cruzi* by a mechanism that relies on cathepsin-dependent caspase-1 cleavage

**DOI:** 10.3389/fimmu.2023.1282856

**Published:** 2023-12-06

**Authors:** Marcelo Pires Amaral, Felipe Daniel Cardoso, Ingrid Sancho de Farias, Rafael Queiroz de Souza, Kely Catarine Matteucci, Ana Claudia Torrecilhas, Karina Ramalho Bortoluci

**Affiliations:** ^1^ Departamento de Farmacologia, Escola Paulista de Medicina/Universidade Federal de São Paulo (EPM/UNIFESP), São Paulo, SP, Brazil; ^2^ Plataforma de Medicina Translacional, Fundação Oswaldo Cruz (FIOCRUZ), Faculdade de Medicina de Ribeirão Preto (FMRP), Ribeirão Preto, SP, Brazil; ^3^ Departamento de Ciências Farmacêuticas, Instituto de Ciências Ambientais, Químicas e Farmacêuticas, Universidade Federal de São Paulo (UNIFESP), Diadema, SP, Brazil

**Keywords:** NAIP/NLRC4, inflammasome, *Trypanosoma cruzi*, cathepsins, caspase-1

## Abstract

Inflammasomes are large protein complexes that, once activated, initiate inflammatory responses by activating the caspase-1 protease. They play pivotal roles in host defense against pathogens. The well-established role of NAIP/NLRC4 inflammasome in bacterial infections involves NAIP proteins functioning as sensors for their ligands. However, recent reports have indicated the involvement of NLRC4 in non-bacterial infections and sterile inflammation, even though the role of NAIP proteins and the exact molecular mechanisms underlying inflammasome activation in these contexts remain to be elucidated. In this study, we investigated the activation of the NAIP/NLRC4 inflammasome in response to *Trypanosoma cruzi*, the protozoan parasite responsible for causing Chagas disease. This parasite has been previously demonstrated to activate NLRP3 inflammasomes. Here we found that NAIP and NLRC4 proteins are also required for IL-1β and Nitric Oxide (NO) release in response to *T. cruzi* infection, with their absence rendering macrophages permissive to parasite replication. Moreover, *Nlrc4* ^-/-^ and *Nlrp3* ^-/-^ macrophages presented similar impaired responses to *T. cruzi*, underscoring the non-redundant roles played by these inflammasomes during infection. Notably, it was the live trypomastigotes rather than soluble antigens or extracellular vesicles (EVs) secreted by them, that activated inflammasomes in a cathepsins-dependent manner. The inhibition of cathepsins effectively abrogated caspase-1 cleavage, IL-1β and NO release, mirroring the phenotype observed in *Nlrc4* ^-/-^/*Nlrp3* ^-/-^ double knockout macrophages. Collectively, our findings shed light on the pivotal role of the NAIP/NLRC4 inflammasome in macrophage responses to *T. cruzi* infection, providing new insights into its broader functions that extend beyond bacterial infections.

## Introduction

*Trypanosoma cruzi*, the causative agent of Chagas disease, represents a significant global health burden, affecting millions of people primarily in Latin America (1). This intracellular parasite can evade host immune defenses, leading to chronic infection and the development of severe pathological consequences. Unraveling the intricate host-parasite interplay is crucial for the development of effective therapeutic strategies against *T. cruzi* (2, 3).

Inflammasomes, multiprotein complexes assembled in the cell cytosol in response to infections or cytosolic disturbances, act as key regulators of the innate immune response. They are responsible for the inflammatory caspase-1 activation, a cysteine protease that cleaves IL-1β, IL-18, and gasdermin-D (GSDM-D), culminating in cytokine secretion and pyroptosis (4, 5).

The role of the NLRP3 inflammasome during protozoan infections has been extensively demonstrated. NLRP3 is involved in host resistance against *Toxoplasma gondii (* 6), *Plasmodium* spp. (7, 8), *Leishmania* spp. (9, 10), and *T. cruzi (* 11, 12). On the other hand, it has been reported that NLRP3-deficient mice seem to be protected against cerebral (13) and placental malaria (14), as well as the severity of leishmaniasis caused by *L. braziliensis (* 15) and *L. major* Seidman strain (16) infections. NLRP3 seems to be activated by common cytosolic disturbances induced by these parasites, such as cathepsins extravasation (7, 9, 11), reactive oxygen species (ROS) (17–19), potassium (K^+^) efflux (9, 20), calcium (Ca^2+^) influx (21, 22), and crystal accumulation (23).

NAIP/NLRC4 inflammasome activation occurs after recognition of bacterial components by the NLR family apoptosis inhibitory protein (NAIP) followed by NLRC4 recruitment (24, 25). However, several studies have reported the participation of NLRC4 in non-bacterial contexts, such as *Candida albicans (* 26), *Neospora caninum (* 27) and Human immunodeficiency virus (HIV) (28) infections, although the molecular mechanism involved in the inflammasome activation in these contexts remains to be elucidated.

In this study, we unveil a novel role for the NAIP/NLRC4 inflammasome in the context of a pathogenic protozoan parasite. Our research reveals that *T. cruzi* infection activates the NAIP/NLRC4 complex. Subsequently, this activation leads to the regulation of *iNOS* gene expression and secretion of nitric oxide (NO), a well-established trypanocidal molecule (29). Our findings suggest a coordinated action of both NLRP3 and NAIP/NLRC4 inflammasomes, with each playing a non-redundant role in controlling *T. cruzi* infection as evidenced by the strikingly similar responses of *Nlrc4* ^-/-^ and *Nlrp3* ^-/-^ macrophages to *T. cruzi*. Furthermore, our data establishes that the activation of inflammasomes in response to *T. cruzi* is primarily orchestrated by lysosomal cathepsins, without contribution of K^+^ efflux or ROS generation. Lysosomal cathepsins modulate NAIP/NLRC4 and NLRP3 inflammasomes activation during *T. cruzi* infection by interfering with caspase-1 maturation. These findings shed light on the remarkable adaptability of these immune sensor platforms offering novel insights and potential therapeutic targets within the realm of the NAIP/NLRC4 inflammasome in the context of infections.

## Materials and Methods

### Animals and cells

C57BL/6, *Nlrp3* ^-/-^ (30), *Nlrc4* ^-/-^ (31), and *Nlrc4* ^-/-^/*Nlrp3* ^-/-^ mice were purchased from the Center for the Development of Experimental Models for Medicine and Biology (CEDEME) – UNIFESP. *Naip1-7* ^-/-^ and *Nlrc4* ^S533D/S533D^ mice were kindly provided by Dr. Vishva Dixit and Dr. Kim Newton (Genentech Inc). All mice (4-8 weeks old) were housed in a temperature-controlled, free access to water and food, light-cycled facility at UNIFESP.

Peritoneal macrophages (PMs) were obtained by peritoneal lavage 4 days after i.p. of 1% starch solution (Sigma Aldrich) in 2 mL of PBS (w/v). Cells were plated in R3% medium [RPMI 1640 (Gibco), 3% FBS (LGC), 0.16 mM Penicillin (Sigma Aldrich), 0.18 mM Streptomycin (Sigma Aldrich), 12.5 mM HEPES (Sigma Aldrich), 30 mM sodium bicarbonate (Sigma Aldrich), pH 7.2-7.4] and stimulated on the next day.

Bone marrow cells were obtained by femur and tibia flush with cold PBS. Cells were centrifuged, treated with Ammonium-Chloride-Potassium (150 mM NH_4_Cl, 1 mM KHCO_3_, 0.1 mM Na_2_EDTA, pH 7.2-7.4) lysing buffer, washed, centrifuged, and 4.5x10^6^ cells plated in non-adherent T75 flasks in R10% medium [RPMI 1640 (Gibco), 10% FBS (Gibco), 1% Penicillin-Streptomycin (Gibco), 1% GlutaMax (Gibco), 1% HEPES (Gibco), 1% Sodium Pyruvate (Gibco), 1% MEM Non-Essential Amino Acids (Gibco), 1% MEM Vitamin (Gibco), 20% L929 supernatant, and 55 μM β-mercaptoethanol (Sigma Aldrich), pH 7.2-7.4]. Cell medium was replaced on day 3 and 5. On day 7, cells were maintained for 15 minutes in cold PBS 2% EDTA and 2% FBS, collected, plated, and stimulated on the next day.

Cells were infected by *T. cruzi* in a ratio of 5:1 (parasites:cell) for 2 h, then replaced by fresh medium and maintained during all the infection time. When pertinent, cells were treated with cathepsins, K^+^ efflux and ROS pharmacological inhibitors (25 μM CA-074Me, 30 mM KCl and 25 μM Apocynin, respectively) for 1.5 h before the stimuli and maintained during the entire experiment. Alternatively, the CA-074Me dose-response curve experiments were performed using concentrations ranging from 6 μM to 50 μM. For IL-1β secretion positive controls, cells were first primed with LPS (200 ng/mL) for 3 h and then the supernatant was replaced by 3 μg/mL of flagellin (Invivogen) inserted into lipid vesicles (DOTAP) (Sigma Aldrich) for 3 h or 10 μM of nigericin (Invivogen) for 1.5 h.

### Parasites

The *T. cruzi* Y strain from the Dante Pazzanese Institute was cultured in LLC-MK2 cells and frozen in liquid nitrogen. For *in vitro* experiments, the parasites were thawed at 37°C, washed in R3% medium, centrifuged and resuspended in fresh R3% medium before use.

### Parasite load

For the *in vitro* experiments parasite load was checked as previously described (32). Briefly, after each time point supernatant was collected and reserved. The plates were fixed with pure methanol (Merck) or 4% paraformaldehyde (Sigma Aldrich) diluted in PBS for at least 15 min at room temperature. Then the wells were gently washed with warm PBS and incubated with 5 μg/mL DAPI (blue) (Sigma Aldrich) diluted in PBS. At least 6 images of each well were acquired on the IN-Cell Analyzer 2200 equipment, counted, and the average per well was graphically plotted.

### Extracellular vesicles and soluble *T. cruzi* antigens

EVs released from *T. cruzi* were obtained through the centrifugation of trypomastigotes (1000 x g for 15 min). The pellet washes with PBS and subsequently incubated in DMEM medium containing 2% glucose at 37°C with 5% CO_2_ for a duration of 2 h. The parasites were then separated by centrifugation at 1000 x g for 10 min, leading to the collection of approximately 1 mL of supernatant. This supernatant was filtered and diluted 1:2 using 200 mM ammonium acetate (pH 6.5). The quantification of EVs was conducted using the NanoSight NS300 device produced by Malvern Panalytical Ltd, revealing that most particles (> 90%) ranged in size from approximately 138 nm to 230 nm.

For the nanoparticle tracking analysis (NTA), a 500 µL volume of the isolated EVs sample was meticulously introduced into the laser chamber of the NanoSight NS300 apparatus. This injection process was conducted with care to prevent the introduction of air bubbles or any loss of the sample. Readings were taken in triplicate for each sample, with each reading extending for 30 seconds at a frame rate of 25 frames per second. This approach facilitated the real-time tracking and measurement of EVs, capturing their Brownian motion. Subsequently, cells were exposed to a stimulation involving 1x10^8^ particles/well over a period of 48 hours.

In order to obtain soluble antigens, *T. cruzi* at a ratio of 5:1 (parasites:cell) per well were centrifuged at 3200 x g 10 min and resuspended in PBS. The parasites were lysed with 5 cycles of freezing in liquid nitrogen and thawing in a water bath at 37°C, followed by sonication for 30 s at 30%. The homogenate was again centrifuged at 3200 x g 10 min and the supernatant containing soluble *T. cruzi* antigens were diluted for *in vitro* stimulation for 48 h.

### IL-1β, IL-6, LDH and NO measurement

The IL-1β and IL-6 cytokines quantification were performed by collecting the supernatant after each time point and a sandwich ELISA was executed according to the manufacturer’s instructions (Invitrogen). The plates were read at 450 nm absorbance on SpectraMax equipment. The LDH measurement was performed according to the manufacturer’s instructions (Invitrogen) by collecting the supernatant. The plate was read at 680 nm and subtracted by the 490 nm absorbance on SpectraMax equipment. The NO production was assessed indirectly by the measurement of nitrite concentration by *Griess* reaction immediately after the end of each time point. Briefly, 50 μL of Griess reagent (1% sulfanilamide, 0.1% naphthalene diamine dihydrochloride - NEED, 45% CH_3_COOH) was added to 50 μL of supernatant samples and to 50 μL of a serial diluted standard curve. The samples were read at 540 nm absorbance on SpectraMax equipment.

### RT-qPCR

To determine the relative gene expression, cells were infected with *T. cruzi* for 6 h for *iNOS* or 24 h for *Pro-IL-1β, Nlrp3* or *Nlrc4* expression. Briefly, the mRNAs were isolated using the TRIzol (Invitrogen) method. The concentration and purification of mRNAs were analyzed by reading in a NanoDrop 2000c spectrophotometer (ThermoFisher Scientific, Inc). The samples were evaluated in the ratio of 260/280 nm and 260/230 nm absorbance, where only ratios above 1.8 were used, indicating the absence of contaminants. The cDNA was generated from 500 ng of total RNA by M-MLV Reverse Transcriptase (Invitrogen) following the manufacturer’s instructions. The cDNA (50 ng) was homogenized with TaqMan Universal PCR Master Mix (Applied Biosystems). All values ​​were normalized using the expression level of the endogenous control *β-actin* (Mm02619580_g1). Gene expression levels were shown through relative expression analysis. The relative gene expression of *iNOS* in all genotypes was compared to uninfected C57BL/6 cells. Reactions were conducted in a 7500 Real Time PCR system (Applied Biosystems).

### Immunofluorescence ASC specks

Cells were plated for 18 h in a 96-well black plate (Greiner) with a clear bottom for microscopy. On the next day cells were infected with *T. cruzi* as described above and after 4 h the supernatant was removed, and cells were fixed with 4% paraformaldehyde (Sigma Aldrich) diluted in PBS for at least 15 min. Then, wells were washed twice with warm PBS, followed by block/permeabilization buffer [10% BSA (Sigma Aldrich), 1% FBS (LGC), 0.5% Triton-X 100 (Sigma Aldrich), diluted in PBS] for 1 h at room temperature. Wells were carefully washed twice with warm PBS and incubated for 18 h at 4°C with 1:1000 anti-ASC (Millipore, clone 2EI-7). On the next day wells were washed again with warm PBS and incubated with secondary antibody Alexa-fluor 647 (red) (Invitrogen) 1:1000 for 1 h at room temperature. Wells were washed again, incubated with 5 μg/mL DAPI (blue) (Sigma Aldrich) and images were acquired on IN Cell Analyzer 2200 equipment.

### Western blotting

For western blotting assays 1x10^6^ cells were plated, in duplicates, in a 24-well plate and maintained for 18 h at 37°C 5% CO_2_. On the next day cell media was replaced by OptiMem (Gibco) and treated with cathepsins inhibitor if necessary. Then, cells were infected with *T. cruzi* as described above for 24 h. The duplicate of supernatants were collected and combined, then precipitated with methanol and chloroform. While the duplicate cell samples were lysed, collected and combined. Samples were run on a 13.5% polyacrylamide SDS-PAGE, transferred to PVDF membrane (Merck), blocked with 5% BSA (Sigma Aldrich) diluted in TBStween 0.05% for 1 h at room temperature, washed 3x with TBStween 0.05% and incubated for 18 h with 1:500 anti-caspase-1 (Adipogen) at 4°C. The membrane was washed 3x with TBStween 0.05%, incubated with 1:1000 horseradish peroxidase-labeled goat anti-mouse IgG (Santa Cruz) for 1 h at room temperature, washed again, and developed by chemiluminescence using ECL (Santa Cruz) acquired in Alliance 4.7 software (Uvitec; Cambridge). Quantification of caspase-1 p20 bands was performed using ImageJ software.

### Data analysis

Statistical significances (p-values) were calculated by One-way ANOVA followed by Tukey honestly significantly different (HSD) *post hoc* test or Student’s t-test. Data were considered significant when p ≤ 0.0332 (∗), 0.0021 (∗∗), 0.0002 (∗∗∗), or 0.0001 (∗∗∗∗). Statistical analysis and graphical representation were performed using GraphPad Prism version 9.3.0 software.

## Results

### NAIP/NLRC4 inflammasome is required for macrophage trypanocidal activity

**Figure 1:**
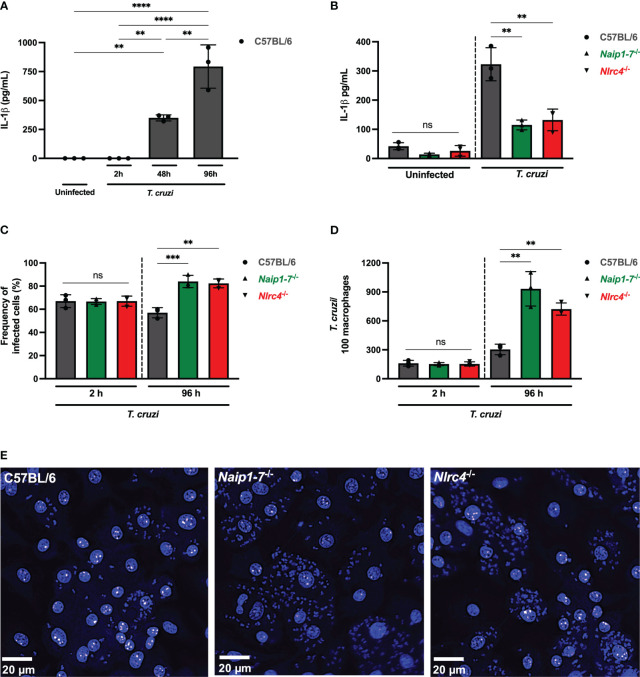
NAIP/NLRC4 inflammasome is activated during *T. cruzi* infection. Elicited PMs from C57BL/6, *Naip1-7* ^-/-^ and *Nlrc4* ^-/-^ mice were plated (5x10^5^/well) in triplicates and on the next day cells were infected by *T. cruzi* Y strain MOI 5:1 (parasites:cell) for 2 h, then supernatant was replaced by fresh R3% medium. After **(A)** 2 h, **(A, B)** 48 h and **(A)** 96 h the supernatant was collected to quantify IL-1β production. Then, the 96 h-infected cells were fixed with methanol for at least 15 min, replaced by DAPI (blue) staining and images were acquired immediately on IN Cell Analyzer 2200. **(C)** Frequency of infected cells. **(D)** Number of parasites/100 macrophages. **(E)** Representative images of *T. cruzi*-infected C57BL/6, *Naip1-7* ^-/-^ and *Nlrc4* ^-/-^ PMs. The experiments were performed at least twice. Statistical significance was calculated by One-way ANOVA followed by Tukey’s *post hoc* test, **p < 0.0021, ***p < 0.0002, ****p < 0.0001, ns, not significant.

NLRC4 activation is well characterized in bacterial contexts (24, 25). Recent studies have reported the involvement of NLRC4 in non-bacterial infections such as *C. albicans*, *N. caninum* and HIV infection (26–28). Given that the involvement of NAIP in most of these studies remains to be elucidated, and the role of NAIP/NLRC4 in response to *T. cruzi* infection is largely unexplored, we decided to investigate *Naip1-7* ^-/-^ and *Nlrc4* ^-/-^ macrophage responses to *T. cruzi* infection. As previously demonstrated by our group (11), *T. cruzi* infection resulted in a time-dependent IL-1β secretion (**Figure 1A**). Notably, *T. cruzi*-infected *Naip1-7* ^-/-^ and *Nlrc4* ^-/-^ PMs exhibited diminished IL-1β response compared to the C57BL/6 wild-type (WT) PMs (**Figure 1B**).

To delve deeper into the potential contribution of the NAIP/NLRC4 inflammasome during *T. cruzi* infection on macrophages, we assessed intracellular parasites count at 2 h (parasite entry) and 96 h (parasite replication load) after infection. At 2 h post-infection the frequency of infected cells (**Figure 1C**) and the number of intracellular parasites (**Figure 1D**) observed in *Naip1-7* ^-/-^ and *Nlrc4* ^-/-^ PMs were similar to the C57BL/6 WT cells, demonstrating that cell invasion by *T. cruzi* was not affected by the absence of NAIP or NLRC4, similar as previously described for NLRP3 (11). However, at 96 h post-infection *Naip1-7* ^-/-^ and *Nlrc4* ^-/-^ PMs were more permissive to *T. cruzi* replication than C57BL/6 WT cells, presenting higher frequency of infected cells (**Figure 1C**) and higher numbers of intracellular amastigotes (**Figures 1D, E**) in comparison to their littermate control cells. These observations underscore the involvement of NAIP/NLRC4 inflammasomes in the macrophages’ ability to counteract the proliferation of *T. cruzi*.

NLRC4 phosphorylation on serine 533 (Ser 533) residue seems to optimize NAIP/NLRC4 inflammasome activation in response to their classical agonists (33). However, the constitutive phosphorylation of NLRC4 did not render macrophages more resistant to *T. cruzi*, since the frequency of infected cells were similar between phosphomimetic mutant *Nlrc4* ^S533D/S533D^ and C57BL/6 WT cells (**Supplementary Figure 1A**) and the amastigote numbers were even higher than those observed in C57BL/6 WT cells (**Supplementary Figures 1B, C**), thus reinforcing that NAIP/NLRC4, but not NLRC4 *phosphorylation*, is required for *T. cruzi* replication control by macrophages.

### Both NLRP3 and NAIP/NLRC4 are required for macrophage responses to *T. cruzi* infection

**Figure 2:**
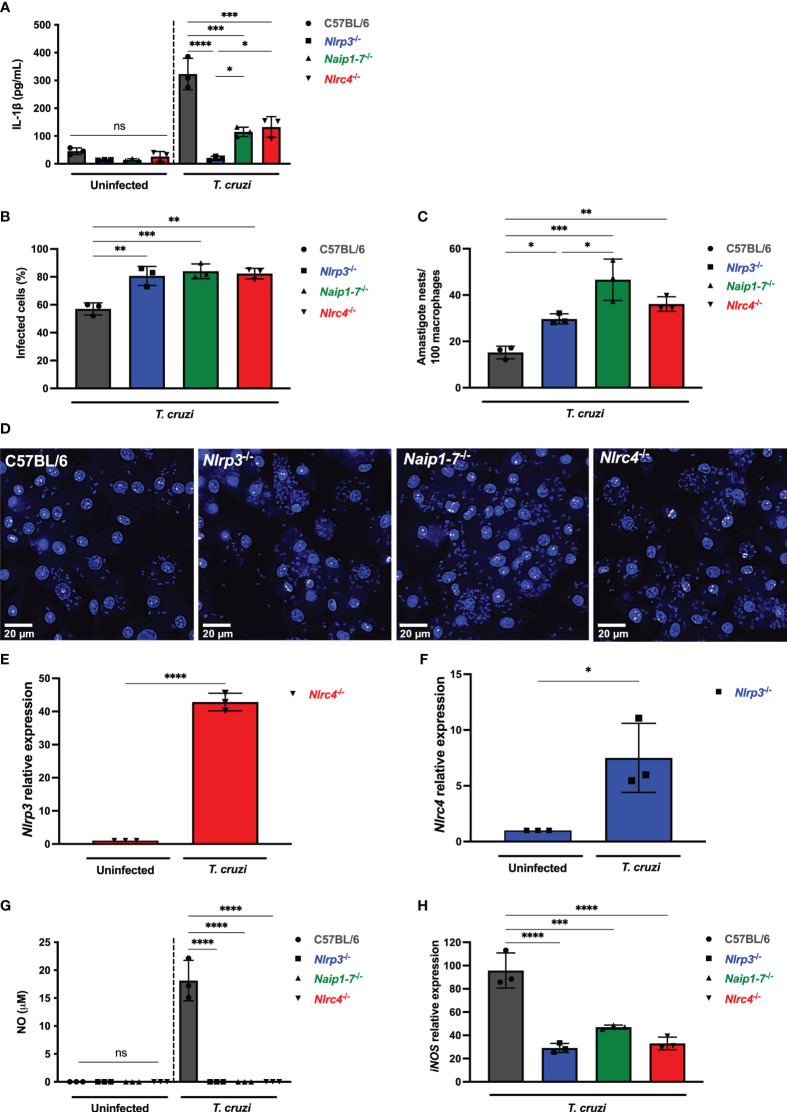
NLRP3 and NAIP/NLRC4 inflammasomes are required for effective macrophage responses to *T. cruzi* infection. **(A–D, G, H)** Elicited PMs from C57BL/6, *Nlrp3* ^-/-^, *Naip1-7* ^-/-^ and *Nlrc4* ^-/-^ mice were plated (5x10^5^/well) in triplicates and on the next day cells were infected by *T. cruzi* Y strain MOI 5:1 (parasites:cell) for 2 h, then supernatant was replaced by fresh R3% medium. After **(A)** 48 h the supernatant was collected to quantify IL-1β production. **(B–D)** 96 h post-infection cells were fixed with methanol for at least 15 min, replaced by DAPI (blue) staining and images were acquired immediately on IN Cell Analyzer 2200. **(B)** Frequency of infected cells. **(C)** Parasite burden. Amastigote nests represent at least 15 parasites/nest. **(D)** Representative images of *T. cruzi*-infected C57BL/6, *Nlrp3* ^-/-^, *Naip1-7* ^-/-^ and *Nlrc4* ^-/-^ PMs. BMDM from *Nlrc4* ^-/-^ and *Nlrp3* ^-/-^ mice were plated (2x10^5^/well), infected on the next day by *T. cruzi* Y strain MOI 5:1 (parasites:cell) for 2 h, then supernatant was replaced by fresh R3% medium and incubated for 24 h. The mRNA was extracted and the **(E)** *Nlrp3* and **(F)** *Nlrc4* relative gene expression to the paired uninfected cells were quantified by RT-qPCR. **(G)** The NO production and **(H)** *iNOS* expression (RT-qPCR) were quantified after 48 h and 6 h, respectively, after *T. cruzi* Y strain infection. The *iNOS* relative gene expression was compared to uninfected C57BL/6 cells. The experiments were performed at least twice. Statistical significance was calculated by **(A–C, G, H)** One-way ANOVA followed by Tukey’s *post hoc* test or **(E, F)** Student’s *t*-test, *p < 0.0332, **p < 0.0021, ***p < 0.0002, ****p < 0.0001, ns, not significant.

We have previously demonstrated the involvement of NLRP3 inflammasome in controlling *T. cruzi* replication (11). To evaluate the relative roles of NLRP3 and NAIP/NLRC4 inflammasomes, we compared the responses of *Nlrp3* ^-/-^, *Naip1-7* ^-/-^ and *Nlrc4* ^-/-^ macrophages to *T. cruzi*. PMs presented reduced IL-1β secretion after *T. cruzi* infection when NLRP3, NAIP or NLRC4 proteins were absent (**Figure 2A**). Importantly, these macrophages responded as expected to nigericin and flagellin, the classical stimuli of NLRP3 and NAIP/NLRC4 inflammasomes, respectively (**Supplementary Figure 2A**). Similar results during *T. cruzi* infection were observed in bone marrow-derived macrophages (BMDM) (**Supplementary Figure 2B**).

Remarkably, *Nlrp3* ^-/-^, *Naip1-7* ^-/-^ and *Nlrc4* ^-/-^ macrophages were similarly permissive to *T. cruzi* replication with higher frequency of infected cells (**Figure 2B**) and intracellular amastigotes (**Figures 2C, D**) in comparison to C57BL/6 WT cells. Of note, *T. cruzi* infection resulted in a 40-times increase of *Nlrp3* expression in *Nlrc4* ^-/-^ macrophages (**Figure 2E**), although a less robust increase of *Nlrc4* expression was observed in *Nlrp3* ^-/-^ cells (**Figure 2F**). Thus, the elevated NLRP3 expression alone was insufficient to rescue the impairment of *Nlrc4* ^-/-^ cells in controlling *T. cruzi* replication (**Figures 2C, D**), emphasizing once again the requirement of NLRC4 inflammasome for the macrophages’ trypanocidal capacity.

We have previously demonstrated the role of NLRP3-mediated NO secretion, an important trypanocydal molecule (11, 29). Here our findings revealed that the permissiveness of *Naip1-7* ^-/-^ and *Nlrc4* ^-/-^ macrophages to *T. cruzi* replication was correlated to the impairment of NO secretion, similar as found for *Nlrp3* ^-/-^ cells (**Figure 2G**). Furthermore, NLRP3, NAIP and NLRC4 seemed to control NO secretion through the transcriptional regulation of *iNOS* expression (**Figure 2H**). Altogether, these results demonstrate a non-compensatory role of NLRP3 and NAIP/NLRC4 inflammasomes in macrophage responses to *T. cruzi* infection.

### Inflammasome activation by *T. cruzi* requires live parasite and relies on lysosomal cathepsins

**Figure 3:**
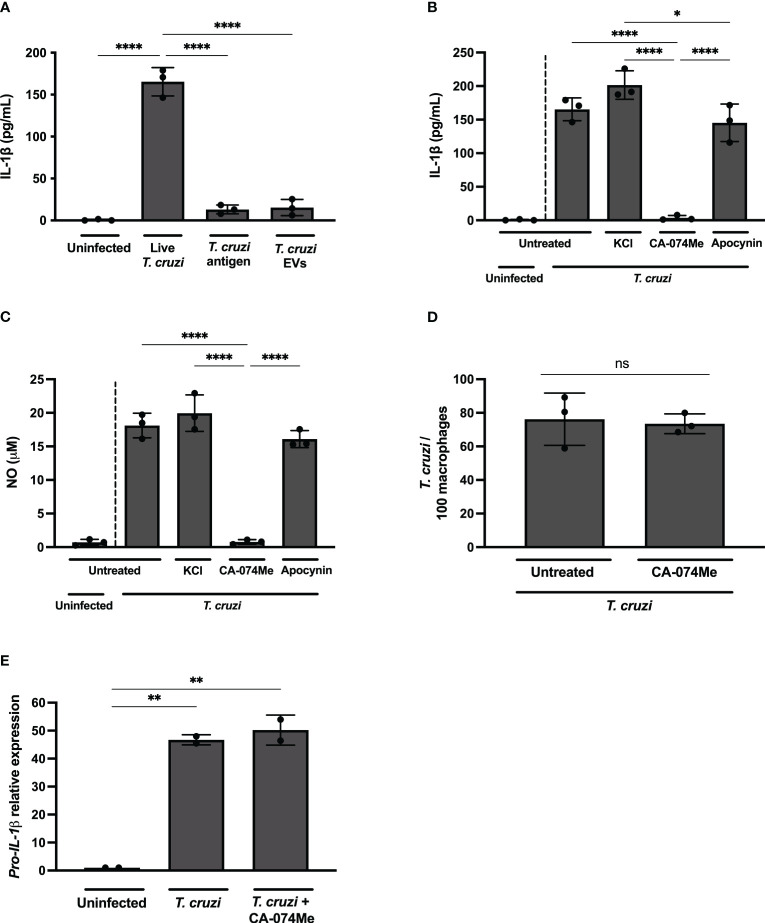
Live *T. cruzi* trypomastigotes activate inflammasomes in a cathepsin-dependent manner. Elicited PMs from C57BL/6 mice were plated (5x10^5^/well) and on the next day cells were infected with *T. cruzi* Y strain MOI 5:1 (parasites:cell) for 2 h, then supernatant was replaced by fresh R3% medium for **(A–C)** 48 h, **(D)** 2 h or **(E)** 24 h. **(A)** Alternatively, cells were only incubated with *T. cruzi* soluble antigens or 1x10^8^ EVs/well for 48 h. **(B–D)** Also, cells were pre-treated or not with KCl (30 mM), CA-074Me (25 μM) and Apocynin (25 μM) for 1.5 h prior to the infection and maintained during the entire experiment. The supernatant was collected and cells were fixed with methanol for at least 15 min when necessary. **(A, B)** IL-1β and **(C)** NO production. **(D)** Prevalence of *T. cruzi* infection. **(E)** The mRNA was extracted and the relative *Pro-IL-1β* gene expression was quantified by RT-qPCR and compared to the paired uninfected cells. The experiments were performed at least twice. Statistical significance was calculated by **(A–C, E)** One-way ANOVA followed by Tukey’s *post hoc* test or **(D)** Student’s *t*-test, *p < 0.0332, **p < 0.0021, ****p < 0.0001, ns, not significant.

Since canonical activation of NLRC4 requires the recognition of bacterial ligands by NAIP, we next evaluated if molecules expressed or secreted by *T. cruzi* were able to activate NAIP/NLRC4 inflammasome. Soluble antigens or EVs secreted by *T. cruzi* did not induce IL-1β secretion by C57BL/6 WT PMs, unlike live trypomastigotes (**Figure 3A**). This data suggests that NAIP/NLRC4 inflammasome activation by *T. cruzi* could be a response to the cytosolic disturbances caused by the parasite infection, similar as observed for NLRP3.

While K^+^ efflux and ROS inhibition, known pathways associated with NLRP3 activation (5, 34), did not exert any discernible influence on IL-1β (**Figure 3B**) or NO (**Figure 3C**) release in *T. cruzi*-infected macrophages, a distinct outcome was observed upon cathepsins inhibition. The inhibition of lysosomal cathepsins, protease enzymes implicated in both NLRP3 (35) and NLRC4 (36, 37) activation, abrogated IL-1β and NO secretion in response to *T. cruzi* infection (**Figures 3B, C**). As expected, CA-074Me and KCl, but not NaCl or Apocynin, significantly reduced IL-1β secretion by nigericin-stimulated macrophages (**Supplementary Figure 2C**). Furthermore, cathepsins inhibition also reduced IL-1β secretion in macrophages stimulated with flagellin, classical activator of NAIP/NLRC4 inflammasomes (**Supplementary Figure 2D**). These results demonstrate the effect of cathepsins on both NLRP3 and NAIP/NLRC4-mediated responses.

CA-074Me inhibited NO (**Supplementary Figure 3A**) and IL-1β (**Supplementary Figure 3B**) but not IL-6 (**Supplementary Figure 3C**) in response to *T. cruzi* infection in a dose-dependent manner, ruling out off-target actions of this inhibitor. Furthermore, there was no cellular cytotoxicity observed in CA-074Me-treated cells, as evidenced by the absence of statistically significant differences in LDH release, even at high concentrations of the chemical compound (**Supplementary Figure 3D**).

Of note, the number of intracellular parasites was similar at 2 h post infection in both CA-074Me-treated and untreated cells (**Figure 3D**), ruling out any impairment of *T. cruzi* invasion when cathepsins are inhibited. Moreover, the *Pro-IL-1β* expression was unaffected by CA-074Me, suggesting that cathepsins participate in the effector phase of inflammasome activation in response to *T. cruzi* infection, rather than during the priming phase (**Figure 3E**).

### Cathepsins inhibition affects both NLRP3 and NLRC4 inflammasomes by interfering with caspase-1 cleavage in response to *T. cruzi* infection

**Figure 4:**
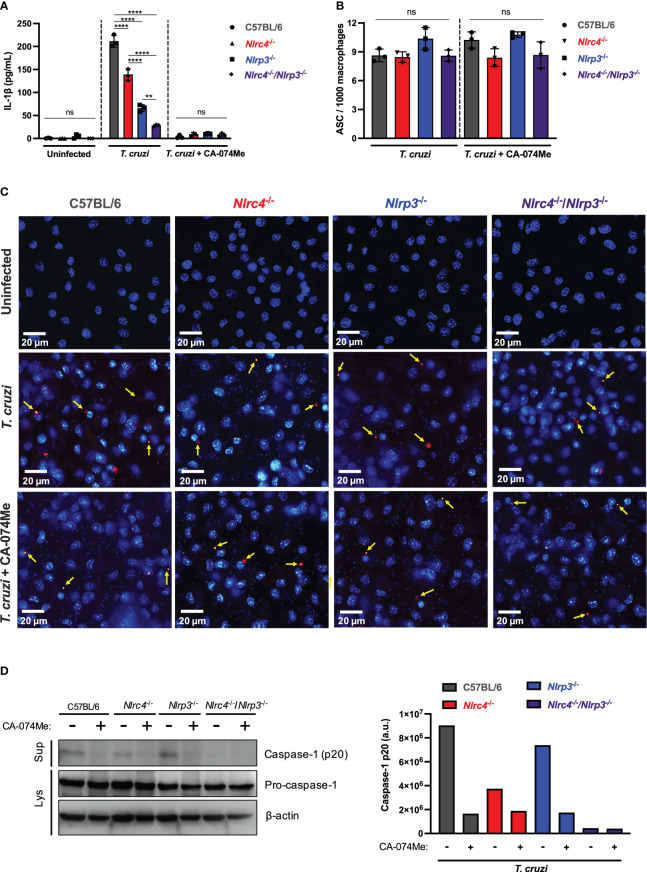
Cathepsins regulate inflammasomes activation during *T. cruzi* infection by modulating caspase-1 maturation. BMDM from C57BL/6, *Nlrc4* ^-/-^, *Nlrp3* ^-/-^ and *Nlrc4* ^-/-^/*Nlrp3* ^-/-^ mice were plated (2x10^5^/well, or 1x10^6^/well for western blotting assay) and on the next day cells were infected by *T. cruzi* Y strain MOI 5:1 (parasites:cell) for 2 h, then supernatant was replaced by fresh R3% medium for **(A)** 48 h and **(B, C)** 4 h, or OptiMem medium for **(D)** 24 h. When pertinent, cells were pre-treated with CA-074Me (25 μM) for 1.5 h prior to the infection and maintained during the entire experiment. **(A)** IL-1β quantification in culture supernatants. **(B)** Frequency of ASC specks. **(C)** Representative images obtained with IN Cell Analyzer 2200 of uninfected, *T. cruzi*- or *T. cruzi*+CA-074Me-infected C57BL/6, *Nlrc4* ^-/-^, *Nlrp3* ^-/-^ and *Nlrc4* ^-/-^/*Nlrp3* ^-/-^ macrophages. Yellow arrows indicate ASC speck. **(D)** Western blot assay for cleaved caspase-1 (p20) (culture supernatants: Sup), and *pro-caspase-1* and β-actin (cell lysates: Lys). Caspase-1 p20 bands quantification was performed using ImageJ software. The experiments were performed at least twice. Statistical significance was calculated by One-way ANOVA followed by Tukey’s *post hoc* test, **p < 0.0021, ****p < 0.0001, ns, not significant.

Since cathepsins inhibition impaired IL-1β secretion, but not *Pro-IL-1β* expression in response to *T. cruzi* infection, we aimed to investigate how cathepsins regulated the inflammasome cascade in this context. First, we observed that inhibiting cathepsins with CA-074Me in *Nlrc4* ^-/-^ or *Nlrp3* ^-/-^ macrophages resulted in a minimal IL-1β response, mimicking the phenotype observed in treated and untreated *Nlrc4^-/-^*/*Nlrp3^-/-^* macrophages (**Figure 4A**).

In order to assess whether cathepsins interfere with inflammasomes assembly in response to *T. cruzi*, we analyzed the frequency of ASC speck formation in macrophages. The frequency of cells expressing ASC specks did not differ among C57BL/6 WT, *Nlrc4* ^-/-^, *Nlrp3* ^-/-^ and *Nlrc4* ^-/-^/Nlrp3^-/-^ macrophages after *T. cruzi* infection (**Figures 4B, C**). Moreover, cells from all strains maintained similar numbers of ASC puncta when treated with CA-074Me (**Figures 4B, C**), suggesting that cathepsins act downstream inflammasomes assembly in response to *T. cruzi* infection.

Next, we investigated the role of cathepsins on caspase-1 maturation. As expected, cleaved caspase-1 (p20) in response to *T. cruzi* was reduced in the absence of NLRC4, and completely abrogated in cells lacking both NLRC4 and NLRP3 (**Figure 4D**). Furthermore, the inhibition of cathepsins abrogated caspase-1 activation in all genotypes, resembling the phenotype observed in *Nlrc4* ^-/-^/Nlrp3^-/-^ cells, thus demonstrating that cathepsins interfered with NLRP3- and NLRC4-dependent caspase-1 maturation during *T. cruzi* infection (**Figure 4D**). Notably, the *pro-caspase-1* expression was not affected by cathepsins inhibition (**Figure 4D**), reinforcing the role of cathepsins during the effector phase of NLRC4 and NLRP3 inflammasomes activation in response to *T. cruzi* infection, rather than during the priming phase.

**Figure 5:**
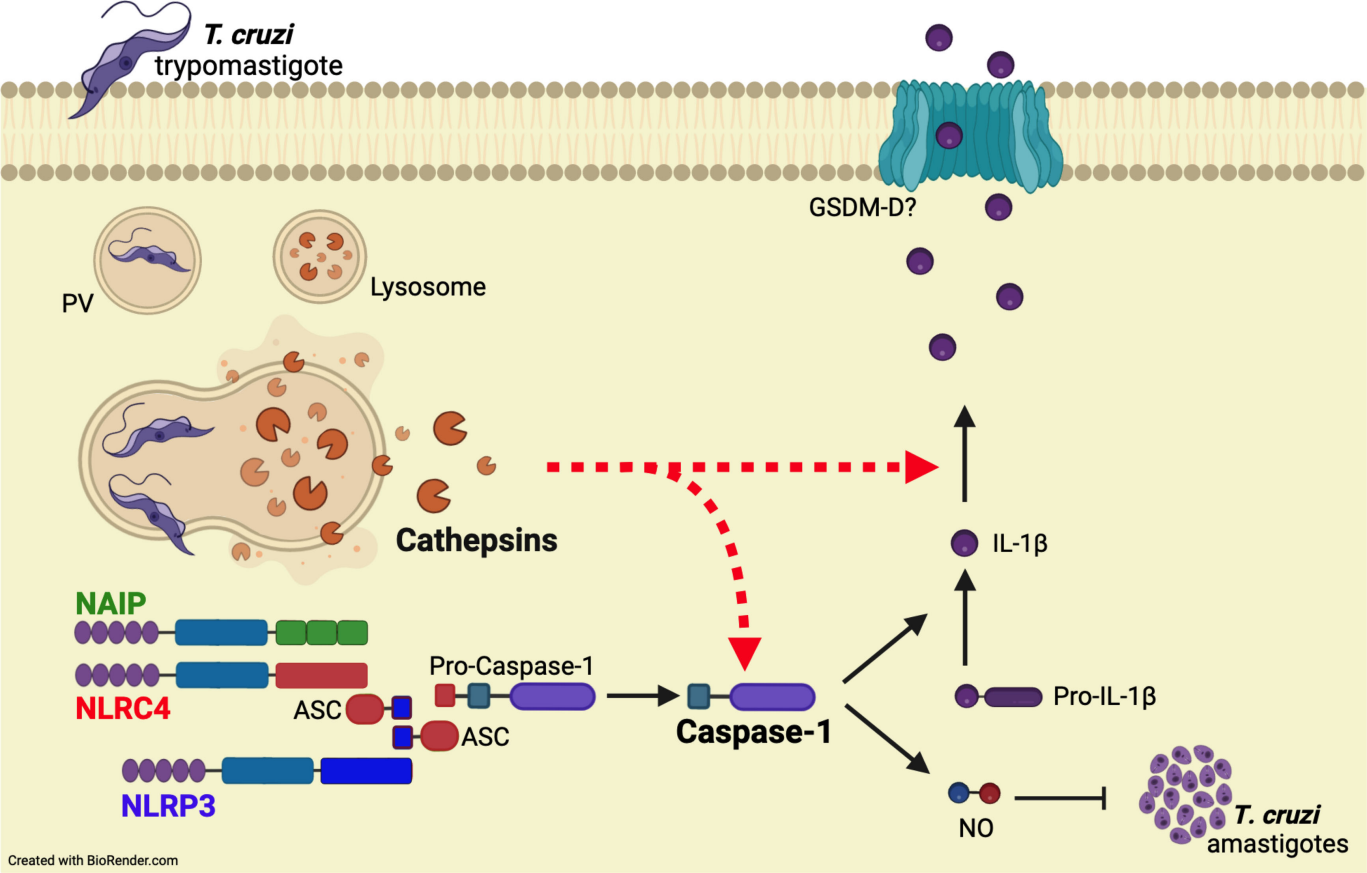
Cathepsins orchestrate the responses of both NAIP/NLRC4 and NLRP3 inflammasomes to *T. cruzi* by interfering with caspase-1 maturation. When *T. cruzi* trypomastigotes infect the host cell, they are initially confined within a parasitophorous vacuole (PV), which subsequently fuses with lysosomes. Following this, the protozoan parasites escape into the cytosol and differentiate into amastigotes for replication. The leakage of cathepsins after lysosomal membrane permeabilization (LMP) orchestrate NAIP/NLRC4 and NLRP3 inflammasome responses to live *T. cruzi* trypomastigotes, leading to caspase-1 cleavage and the consequent release of IL-1β and NO. Accordingly, the inhibition of cathepsins results in the abrogation of these effector responses, resembling the phenotype of double knockout macrophages (*Nlrc4^-/-^/Nlrp3^-/-^*), although the precise mechanisms by which cathepsins modulate caspase-1 maturation remain to be elucidated.

Overall, our results support the concurrent activation of NAIP/NLRC4 and NLRP3 inflammasomes during *T. cruzi* infection. Importantly, cathepsins orchestrate these inflammasome responses by interfering with caspase-1 cleavage and the subsequent IL-1β maturation and NO secretion (**Figure 5**).

## Discussion

Inflammasomes, pivotal components of the innate immune system, have garnered significant attention for their role in host defense against various pathogens. While much has been uncovered about inflammasome assembly, activation, and effector mechanisms, there are still many questions that remain unanswered (4, 5). Among the known inflammasomes, NLRP3 has been the most extensively studied due to its versatile activation by both sterile and non-sterile stimuli (34, 38). Whereas the mechanisms governing NAIP/NLRC4 inflammasome are better elucidated, primarily in the context of bacterial infections (24, 25, 39). However, recent findings have expanded the role of NLRC4 into non-bacterial (26–28) and sterile (40–50) conditions, although the molecular regulation of NAIP/NLRC4 under these contexts remains to be elucidated. In this study, we demonstrated a previously unknown role of NAIP/NLRC4 inflammasome in response to the *T. cruzi* infection, a protozoan parasite responsible for causing Chagas disease. Interestingly, NAIP/NLRC4 activation by *T. cruzi* requires live parasites and relies on lysosomal cathepsins, similar as previously described for NLRP3 (11).

NAIP/NLRC4 inflammasome activation during bacterial infection occurs after recognition of their ligands by NAIP proteins (24, 25). Murine cells express seven NAIP proteins, while human cells have only one. Murine NAIP5 and NAIP6 bind to flagellins from distinct bacteria species, whereas NAIP1 and NAIP2 interact with the proteins from the bacterial type III secretion system (T3SS) (24, 25, 51, 52). NAIP3, NAIP4 and NAIP7 have no clear function so far. Human NAIP (hNAIP) covers all the murine NAIP functions (53). In addition to the direct interaction between NAIP and bacterial agonists, NLRC4 phosphorylation on serine 533 (Ser 533) residue seems to optimize NAIP/NLRC4 inflammasome activation (33). Here, we demonstrated that *Naip1-7* ^-/-^ and *Nlrc4* ^-/-^ macrophages were more permissive to *T. cruzi* replication than C57BL/6 WT cells. In contrast, macrophages from phosphomimetic mutant *Nlrc4* ^S533D/S533D^ mice, that harbors a constitutive phosphorylation on NLRC4 Ser 533, did not demonstrate any resistance to the infection. This highlights the pivotal role of NAIP, rather than NLRC4 phosphorylation, in mediating NLRC4-dependent control of *T. cruzi* by macrophages.

The participation of NAIP in non-bacterial infection was also demonstrated in HIV-1-infected human monocyte-derived macrophages, in which hNAIP was activated by HIV-1 glycoprotein 41 (gp41) leading to the NLRC4 recruitment and IL-18 secretion (28). Although the molecular mechanism of NAIP activation by *T. cruzi* remains to be elucidated, it seems not to involve the interaction with soluble ligands from parasites neither EV secreted by them, since the secretion of IL-1β was only observed in the presence of live trypomastigotes. Accordingly, a previous study demonstrated that irradiated- or heat-killed-*T. cruzi* lost the ability to induce IL-1β secretion in BMDM (12). Therefore, these findings support the hypothesis that inflammasome activation requires cytosolic disturbances induced by live trypomastigotes.

A proposed pathway for NAIP/NLRC4 activation under both infectious and sterile stimuli involves its interaction with other inflammasomes, particularly NLRP3 (27, 41, 42, 54–57). While the precise nature of this interaction is still a subject of debate (55), it has been suggested that the NAIP/NLRC4/ASC complex formed after *Salmonella* infection may recruit NLRP3, potentially amplifying caspase-1 cleavage and subsequent IL-1β cytokine release (42, 54, 57). Although the exact nature of the interaction between NAIP/NLRC4 and NLRP3 within the same inflammasome complex during *T. cruzi* infection requires further elucidation, our results demonstrated that macrophages lacking NLRP3, NAIP1-7, or NLRC4, all exhibited similar deficiencies in controlling parasite replication. This observation suggests a non-redundant role for both NLRP3 and NAIP/NLRC4 inflammasomes in enhancing the macrophage’s trypanocidal capabilities, similar as previously described for macrophage responses to short noncoding retrotransposons (42).

The susceptibility of *Nlrp3* ^-/-^, *Naip1-7* ^-/-^, and *Nlrc4* ^-/-^ macrophages to *T. cruzi* replication was associated with impaired NO secretion. NO is a well-established trypanocidal molecule (11, 29), and we have previously demonstrated the role of NLRP3-induced NO secretion in controlling *T. cruzi* replication in macrophages (11) and microglia (58). Interestingly, even in the presence of a 40-fold increase in *Nlrp3* expression, *Nlrc4* ^-/-^ macrophages failed to release NO, highlighting the participation of the NLRC4 inflammasome in response to *T. cruzi*. NLRC4 has been shown to induce NO secretion in response to cytosolic flagellin by epigenetically regulating *Nos2* through the cleavage of poly [ADP-ribose] polymerase1 (PARP1) by caspase-1 (59, 60). This is in line with our observations regarding the requirement of NLRP3, NAIP, and NLRC4 in regulating *Nos2* expression at the transcriptional level in response to *T. cruzi*.

Interestingly, it appears that both NAIP/NLRC4 and NLRP3 inflammasomes are regulated by lysosomal cathepsins during *T. cruzi* infection. This is substantiated by the fact that pharmacological inhibition of these cysteine proteases effectively halted IL-1β secretion, NO release, and caspase-1 cleavage, whereas inhibition of K^+^ efflux or ROS, had no such impact. These outcomes parallel the effects observed when both NLRP3 and NLRC4 proteins were genetically deleted. Lysosomal membrane permeabilization (LMP) and the consequent leakage of cathepsins into the cytosol are known pathways associated with NLRP3 activation in response to different stimuli (35, 61, 62), including protozoan infections (7, 9, 11). Recent studies have further highlighted the role of cathepsins in regulating NAIP/NLRC4-dependent IL-1β secretion by murine and human macrophages in response to cytosolic flagellin (37).

Several mechanisms have been proposed for cathepsin-mediated inflammasome activation. Cathepsins can influence ASC speck formation, caspase-1 maturation, *pro-IL-1β* transcription, and IL-1β secretion in response to classical NLRP3 agonists like particulate material, ATP, or toxins (35, 63, 64). In response to flagellin, a well-known NLRC4 agonist, cathepsins seem to collaborate with gasdermin D (GSDMD) to enhance IL-1β secretion (37). This study, however, suggests that cathepsins likely do not play a role during the priming and assembly stages of NLRP3 and NAIP/NLRC4 inflammasomes in response to *T. cruzi*. This conclusion is further substantiated by the fact that CA-074Me treatment did not affect the transcription of *pro-IL-1β* and *pro-caspase-1* or the formation of ASC specks. Nonetheless, the presence of CA-074Me effectively inhibited caspase-1 cleavage, aligning with the observations noted in macrophages lacking both NLRC4 and NLRP3. These findings provide compelling evidence that cathepsins orchestrate the activation of both NAIP/NLRC4 and NLRP3 inflammasomes in response to *T. cruzi* infection.

Considering that *T. cruzi* actively recruits lysosomes during invasion, resulting in the formation of the parasitophorous vacuole (PV) (65, 66), it is reasonable to hypothesize that the leakage of lysosomal cathepsins, signaling the parasite’s intrusion into host cells, might represent a pivotal event triggering inflammasome activation during *T. cruzi* infection in macrophages. This novel insight into the interplay between *T. cruzi* and inflammasomes highlights the dual involvement of both NAIP/NLRC4 and NLRP3 inflammasomes in generating effector molecules necessary for controlling *T. cruzi* infection. It sheds light on the intricate functional mechanisms of inflammasomes in the context of infectious diseases, underscoring their significance as potential targets for therapeutic interventions and drug development.

## Data availability statement

The original contributions presented in the study are included in the article/**Supplementary Material**. Further inquiries can be directed to the corresponding author.

## Ethics statement

The animal study was approved by Comitê de Ética no Uso de Animais da UNIFESP (Institutional Animal Care and Use Committees from UNIFESP). The study was conducted in accordance with the local legislation and institutional requirements.

## Author contributions

MA: Conceptualization, Data curation, Formal Analysis, Investigation, Methodology, Validation, Visualization, Writing – original draft, Writing – review & editing. FC: Data curation, Formal Analysis, Investigation, Methodology, Validation, Writing – review & editing. IF: Investigation, Methodology, Validation, Writing – review & editing. RS: Investigation, Writing – review & editing. KM: Methodology, Writing – review & editing. AT: Methodology, Resources, Writing – review & editing. KB: Conceptualization, Funding acquisition, Methodology, Project administration, Resources, Supervision, Validation, Writing – review & editing.

## Supplementary material

Supplementary Figure 1 Constitutive NLRC4 phosphorylation does not improve *T. cruzi* replication restrain. Elicited PMs from C57BL/6 and *Nlrc4* ^S533D/S533D^ mice were plated (5x10^5^/well) in triplicates and on the next day cells were infected by *T. cruzi* Y strain MOI 5:1 (parasites:cell) for 2 h, then supernatant was replaced by fresh R3% medium. After 96 h the supernatant was collected and cells were fixed with methanol for at least 15 min, replaced by DAPI (blue) staining and images were acquired immediately on IN Cell Analyzer 2200. **(A)** Frequency of infected cells. **(B)** Prevalence of *T. cruzi* infection. **(C)** Representative images of *T. cruzi*-infected C57BL/6 and *Nlrc4* ^S533D/S533D^ PMs. The experiments were performed at least twice. Statistical significance was calculated by Student’s *t*-test, *p < 0.0332, ns, not significant.

Supplementary Figure 2 NLRP3 and NAIP/NLRC4 inflammasomes respond to *T. cruzi* infection and classical agonists. **(A, D)** Elicited PMs and **(B, C)** BMDM from C57BL/6, *Naip1-7* ^-/-^, *Nlrp3* ^-/-^ and *Nlrc4* ^-/-^ mice were plated in a density of **(A, D)** 5x10^5^/well and **(B, C)** 2x10^5^/well. **(A)** On the next day PMs were primed with LPS (200 ng/mL) for 3 h and then supernatant was replaced by 10 μM of nigericin for 1.5 h or 3 μg/mL of flagellin inserted into lipid vesicles (DOTAP) for 3 h. The supernatant was collected and IL-1β cytokine was quantified by ELISA. Alternatively, **(B)** BMDM were infected by *T. cruzi* Y strain MOI 5:1 (parasites:cell) for 2 h, then supernatant was replaced by fresh R3% medium. After 48 h the supernatant was collected to quantify IL-1β. The experiments were performed at least three times. **(C, D)** When pertinent, cells were pre-treated and maintained during the entire experiment with KCl (30 mM), CA-074Me (25 μM), Apocynin (25 μM) and NaCl (30 mM) for 1.5 h, primed with LPS (200 ng/mL) for 3 h, and then the supernatant was replaced by **(C)** 10 μM of nigericin for 1.5 h or **(D)** 3 μg/mL of flagellin inserted into lipid vesicles (DOTAP) for 3 h. The supernatant was collected and IL-1β cytokine was quantified by ELISA. The experiments were performed at least three times. Statistical significance was calculated by One-way ANOVA followed by Tukey’s *post hoc* test, *p < 0.0332, **p < 0.0021, ***p < 0.0002, ****p < 0.0001.

Supplementary Figure 3 CA-074Me inhibits NO and IL-1β in dose-dependent manner without interfering on IL-6 production and cytotoxicity. BMDM from C57BL/6 mice were plated (2x10^5^/well) and on the next day cells were infected by *T. cruzi* Y strain MOI 5:1 (parasites:cell) for 2 h, then supernatant was replaced by fresh R3% medium for 48 h. When pertinent, cells were pre-treated with different doses of CA-074Me for 1.5 h prior to the infection and maintained during the entire experiment. The supernatant was collected to quantify **(A)** NO, **(B)** IL-1β, **(C)** IL-6, and **(D)** LDH. The experiments were performed at least twice. Statistical significance was calculated by One-way ANOVA followed by Tukey’s *post hoc* test, **p < 0.0021, ***p < 0.0002, ****p < 0.0001, ns, not significant.
