# Supplementary figures and images for: NAIP/NLRC4 inflammasome participates in macrophage responses to Trypanosoma cruzi by a mechanism that relies on cathepsin-dependent caspase-1 cleavage
Source: Front Immunol. 2023 Dec 6;14:1282856. doi: 10.3389/fimmu.2023.1282856 (PMC10731265; doi:10.3389/fimmu.2023.1282856)

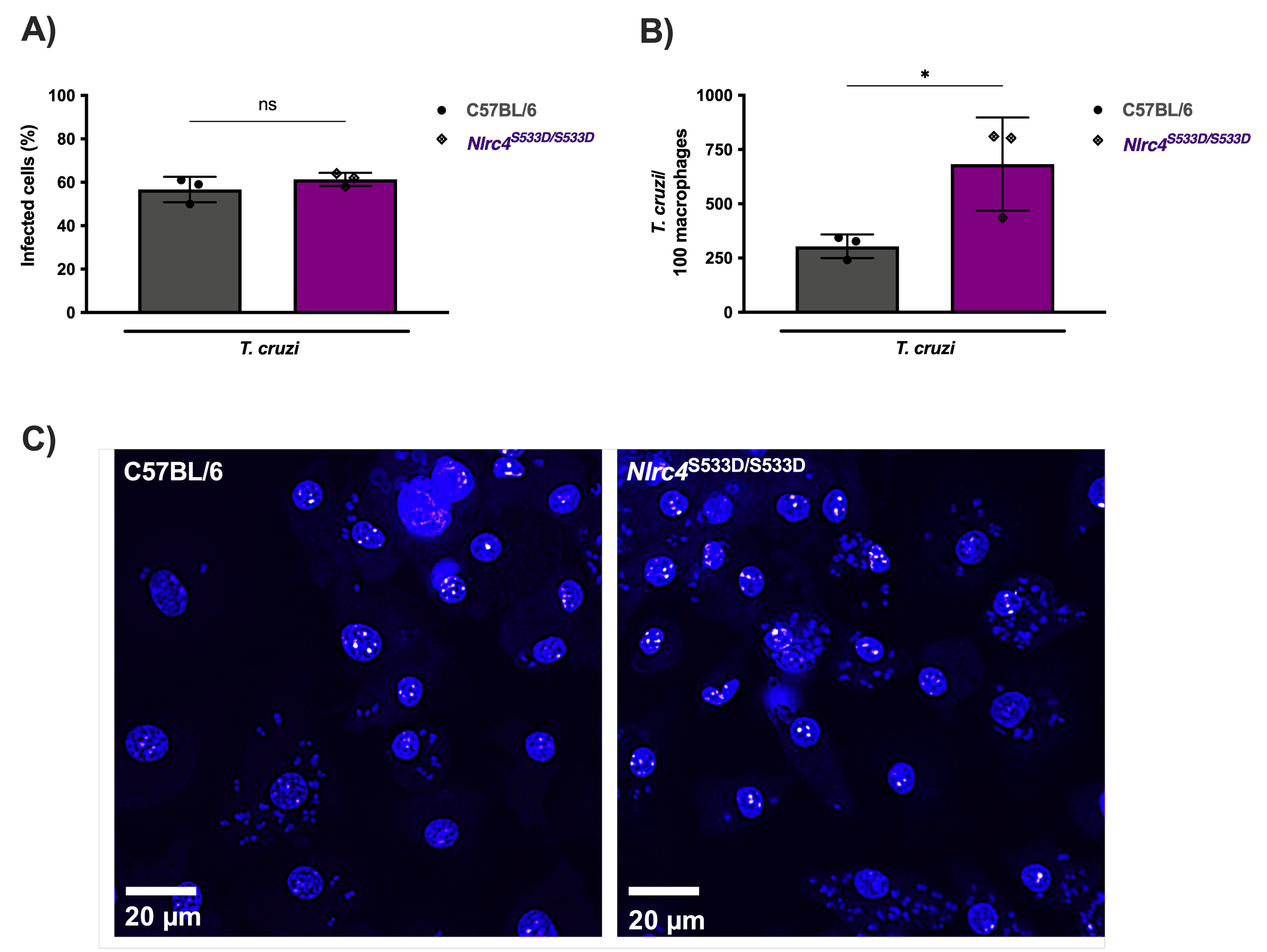

Supplement: Supplementary Figure 1 — Constitutive NLRC4 phosphorylation does not improve T. cruzi replication restrain. Elicited PMs from C57BL/6 and Nlrc4 S533D/S533D mice were plated (5x10^5^/well) in triplicates and on the next day cells were infected by T. cruzi Y strain MOI 5:1 (parasites:cell) for 2 h, then supernatant was replaced by fresh R3% medium. After 96 h the supernatant was collected and cells were fixed with methanol for at least 15 min, replaced by DAPI (blue) staining and images were acquired immediately on IN Cell Analyzer 2200. (A) Frequency of infected cells. (B) Prevalence of T. cruzi infection. (C) Representative images of T. cruzi-infected C57BL/6 and Nlrc4 S533D/S533D PMs. The experiments were performed at least twice. Statistical significance was calculated by Student’s t-test, *p < 0.0332, ns, not significant. [file Image_1.tiff]

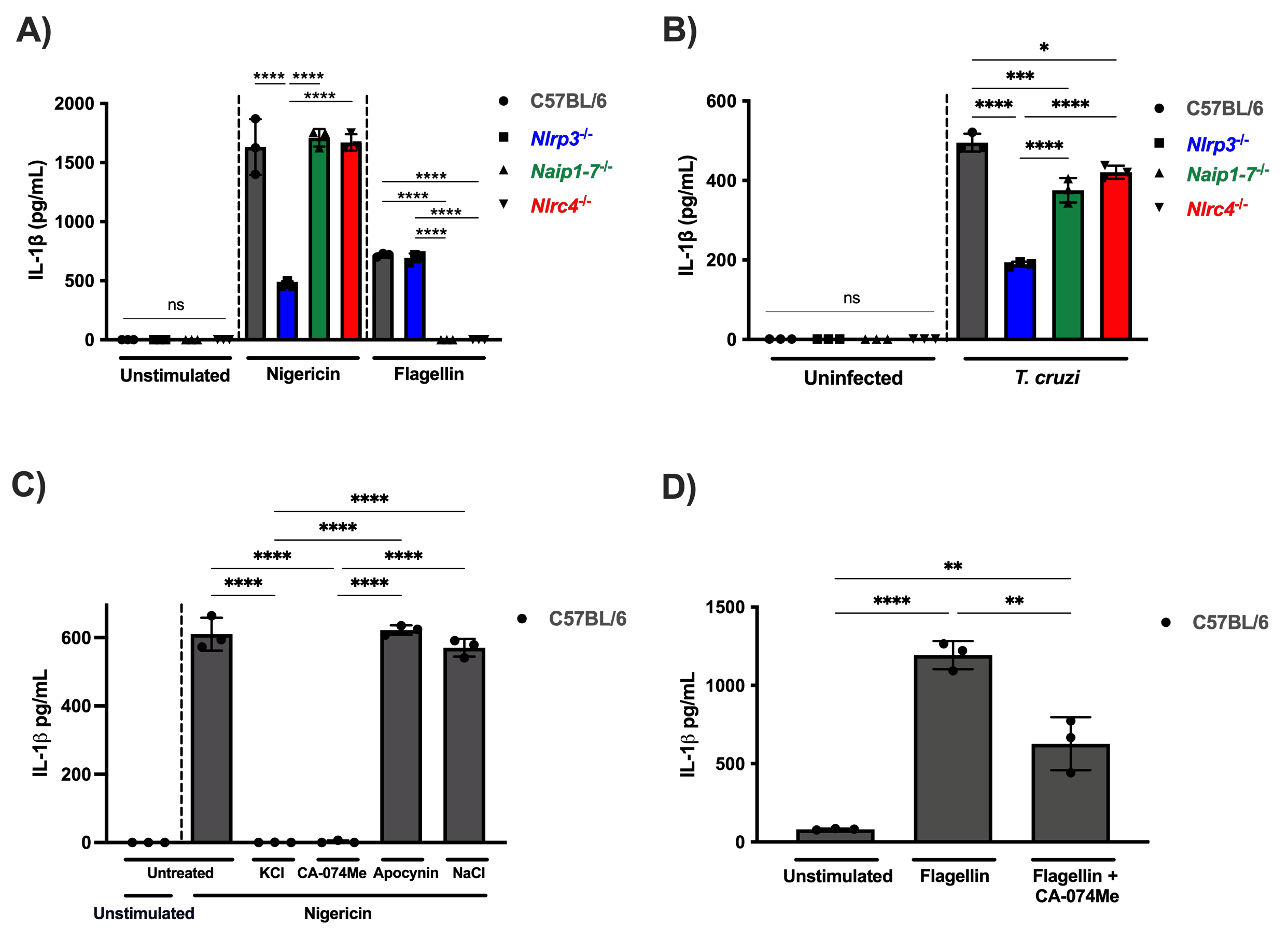

Supplement: Supplementary Figure 2 — NLRP3 and NAIP/NLRC4 inflammasomes respond to T. cruzi infection and classical agonists. (A, D) Elicited PMs and (B, C) BMDM from C57BL/6, Naip1-7 -/-, Nlrp3 -/- and Nlrc4 -/- mice were plated in a density of (A, D) 5x10^5^/well and (B, C) 2x10^5^/well. (A) On the next day PMs were primed with LPS (200 ng/mL) for 3 h and then supernatant was replaced by 10 μM of nigericin for 1.5 h or 3 μg/mL of flagellin inserted into lipid vesicles (DOTAP) for 3 h. The supernatant was collected and IL-1β cytokine was quantified by ELISA. Alternatively, (B) BMDM were infected by T. cruzi Y strain MOI 5:1 (parasites:cell) for 2 h, then supernatant was replaced by fresh R3% medium. After 48 h the supernatant was collected to quantify IL-1β. The experiments were performed at least three times. (C, D) When pertinent, cells were pre-treated and maintained during the entire experiment with KCl (30 mM), CA-074Me (25 μM), Apocynin (25 μM) and NaCl (30 mM) for 1.5 h, primed with LPS (200 ng/mL) for 3 h, and then the supernatant was replaced by (C) 10 μM of nigericin for 1.5 h or (D) 3 μg/mL of flagellin inserted into lipid vesicles (DOTAP) for 3 h. The supernatant was collected and IL-1β cytokine was quantified by ELISA. The experiments were performed at least three times. Statistical significance was calculated by One-way ANOVA followed by Tukey’s post hoc test, *p < 0.0332, **p < 0.0021, ***p < 0.0002, ****p < 0.0001. [file Image_2.tiff]

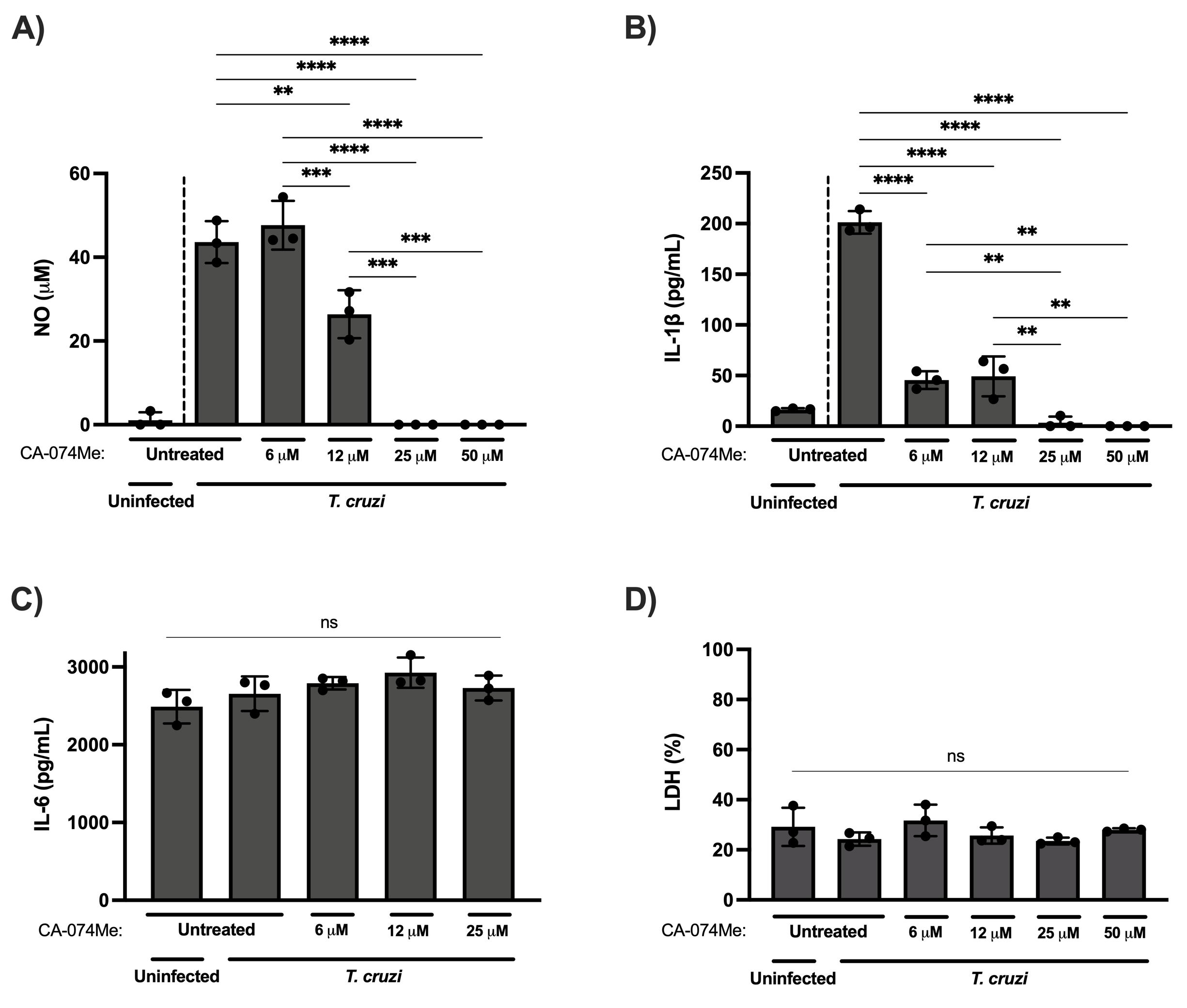

Supplement: Supplementary Figure 3 — CA-074Me inhibits NO and IL-1β in dose-dependent manner without interfering on IL-6 production and cytotoxicity. BMDM from C57BL/6 mice were plated (2x10^5^/well) and on the next day cells were infected by T. cruzi Y strain MOI 5:1 (parasites:cell) for 2 h, then supernatant was replaced by fresh R3% medium for 48 h. When pertinent, cells were pre-treated with different doses of CA-074Me for 1.5 h prior to the infection and maintained during the entire experiment. The supernatant was collected to quantify (A) NO, (B) IL-1β, (C) IL-6, and (D) LDH. The experiments were performed at least twice. Statistical significance was calculated by One-way ANOVA followed by Tukey’s post hoc test, **p < 0.0021, ***p < 0.0002, ****p < 0.0001, ns, not significant. [file Image_3.tiff]
